# Antimicrobial peptide Hs02 with rapid bactericidal, anti-biofilm, and anti-inflammatory activity against carbapenem-resistant *Klebsiella pneumoniae* and *Escherichia coli*

**DOI:** 10.1128/spectrum.01050-24

**Published:** 2024-12-03

**Authors:** Deyi Zhao, Miran Tang, Panjie Hu, Xiaowei Hu, Weijun Chen, Zhexiao Ma, Huanchang Chen, Haifeng Liu, Jianming Cao, Tieli Zhou

**Affiliations:** 1 Department of Clinical Laboratory, Key Laboratory of Clinical Laboratory Diagnosis and Translational Research of Zhejiang Province, The First Affiliated Hospital of Wenzhou Medical University, Wenzhou, China; 2 School of Laboratory Medicine and Life Science, Wenzhou Medical University, Wenzhou, Zhejiang, China; McGill University, Ste-Anne-de-Bellevue, Quebec, Canada

**Keywords:** antimicrobial peptides, Hs02, carbapenem-resistance, LPS neutralization

## Abstract

Carbapenem-resistant *Klebsiella pneumoniae* (CRKP) and *Escherichia coli* (CREC) are frequently detected in clinical settings, restricting the use of carbapenems. Therefore, there is an urgent need for new antimicrobial strategies to address infections caused by CRKP and CREC. This study investigated the antibacterial, anti-biofilm, and anti-inflammatory effects of the cationic antimicrobial peptide Hs02, along with its potential antimicrobial mechanisms against CRKP and CREC. The results revealed that Hs02 had a low minimum inhibitory concentration (MIC) and minimum bactericidal concentration (MBC) against CRKP and CREC, effectively eliminating the bacteria within 30 min. Moreover, Hs02 significantly prevents biofilm formation and disrupts the established biofilms. Further mechanistic studies demonstrated that Hs02 specifically targeted and bound to bacterial outer membrane lipopolysaccharides (LPS), disrupted membrane permeability and integrity, which led to intracellular reactive oxygen species (ROS) accumulation. Furthermore, Hs02 neutralized LPS, thereby suppressing the production of pro-inflammatory cytokines TNF-α, IL-6, and IL-1β in murine macrophage RAW 264.7 cells. *In vitro*, hemolysis and cytotoxicity assays confirmed Hs02’s safety at the tested concentrations and proved that Hs02 improved the survival rate of *Galleria mellonella* larvae. In conclusion, the findings suggest that Hs02’s interaction with LPS and the resulting disruption of membrane integrity may be key factors driving its rapid bactericidal and anti-inflammatory effects.

**IMPORTANCE:**

Eukaryotic antimicrobial peptides are typically amphipathic peptides consisting of approximately 50 amino acids. Many macromolecular proteins in our body contain polypeptide sequences that show characteristics similar to those of antimicrobial peptides. The present research highlights a gap in the current literature regarding the mechanisms by which the intragenic antimicrobial peptide Hs02, derived from human proteins, exerts its rapid bactericidal and anti-inflammatory effects. The findings demonstrate that lipopolysaccharide (LPS) is a key target of Hs02’s antimicrobial activity and that its ability to neutralize LPS is crucial for its anti-inflammatory effects.

## INTRODUCTION

The growing resistance to antimicrobial agents is progressively reducing the available treatment options (1). Drug-resistant pathogens cause 6.22 million fatalities worldwide each year, with bacterial pathogens resistant to conventional antimicrobial drugs accounting for 1.27 million deaths (2). In the latest 2024 WHO Bacterial Priority Pathogens List published by the World Health Organization (WHO), the resistance of *Enterobacteriaceae* to third-generation cephalosporins and carbapenems has been classified as a critical priority. Carbapenems are among the first-line treatment options for infections caused by *Enterobacteriaceae* that are resistant to third-generation cephalosporins. However, the spread of carbapenem-resistant *Klebsiella pneumoniae* (CRKP) and *Escherichia coli* (CREC) has significantly limited the available treatment options, underscoring the urgent need for the development of new antibacterial strategies to address this challenge. (3–6, 7).

Antimicrobial peptides (AMPs) are found in all kinds of living organisms. They may also be synthesized artificially using predicted peptide structures from gene sequences. These peptides generally have a molecular weight between 500 and 10,000 Da and typically consist of 10 or more amino acid residues (8–10). Traditional antimicrobial agents used to treat bacterial infections often cause the breakdown of bacterial cell structures, leading to the release of significant amounts of lipopolysaccharide (LPS). This release triggers an inflammatory response by inducing the release of inflammatory factors (11). In contrast, some AMPs have been shown to neutralize LPS and modulate inflammation-related signaling pathways (12, 13). AMPs also demonstrate clinically useful properties, including fast bactericidal action, anti-biofilm activity, reduced inflammatory response, and synergistic antibacterial activity when combined with other antibacterial agents (14–17).

Biofilm formation decreases drug permeability, leading to bacteria within the biofilm being exposed to sub-inhibitory concentrations of antibiotics, which accelerates the development of bacterial resistance. Research indicates that over 90% of clinical isolates of carbapenemase-producing *Klebsiella pneumonia* (*K. pneumonia*) are moderate to strong biofilm producers (18–21). This indicates that strains with high biofilm production may be more susceptible to developing resistance to carbapenem antibiotics.

Hs02 is an intragenic antimicrobial peptide. It was discovered in the human unconventional myosin 1H protein. This peptide, consisting of 16 amino acid residues, displays amphiphilic α-helical properties in zwitterionic micelles (DPC-d38 micelles) (22). Previous research has demonstrated that Hs02 has antibacterial, anti-biofilm, and anti-inflammatory effects against *Staphylococcus aureus* and *Pseudomonas aeruginosa*. However, the specific targets and mechanisms of its antibacterial activity, as well as how Hs02 inhibits LPS-induced production of macrophage inflammatory cytokines, remain unclear (23).

This study aimed to investigate the multifunctional role of Hs02 in combating CRKP and CREC. The research focused on its rapid bactericidal effects, anti-biofilm and anti-inflammatory activities, and the underlying mechanisms behind its antibacterial and anti-inflammatory properties.

## RESULTS

### Antimicrobial activity and rapid bactericidal effect of antimicrobial peptide Hs02 against CRKP and CREC

An assessment of the antibacterial activity of Hs02, imipenem, meropenem, and ertapenem was carried out using a drug susceptibility test. The table displays that 33 strains of *K. pneumoniae* and *Escherichia coli* (*E. coli*) showed resistance to the three commonly used carbapenems. The minimum bactericidal concentrations (MBC) of Hs02 are also included in the Table 1. The study findings indicated that the minimum inhibitory concentrations (MIC) of Hs02 against CRKP and CREC ranged from 2 to 16 μg/mL, whereas the MBC varied between 4 and 16 µg/mL.

**TABLE 1:** MICs of carbapenems and Hs02 and MBCs of Hs02 against *K. pneumoniae*, *E. coli* with various carbapenemase types and STs^*a*^^,^^*b*^^,^^*c*^

Species	Strains	MIC μg/mL (μM)				MBC μg/mL (μM)	Enzyme types	ST types
		Hs02	MEM	IMP	ETP	Hs02		
*E. coli*	DC5128	4 (2)	64 ^R^	16 ^R^	128 ^R^	4 (2)	NDM	405
	DC6856	4 (2)	16 ^R^	16 ^R^	64 ^R^	8 (4.1)	KPC-2	2003
	DC7114	2 (1)	64 ^R^	16 ^R^	256 ^R^	4 (2)	NDM	410
	DC7914	4 (2)	4 ^R^	8 ^R^	8 ^R^	4 (2)	NDM, TEM	156
	DC8466	4 (2)	4 ^R^	16 ^R^	16 ^R^	4 (2)	NDM	1193
	DC8623	4 (2)	8 ^R^	8 ^R^	32 ^R^	4 (2)	NDM	12531
	DC8647	4 (2)	4 ^R^	8 ^R^	16 ^R^	4 (2)	NDM	1193
	DC10473	4 (2)	8 ^R^	8 ^R^	16 ^R^	4 (2)	KPC-2	602
	DC10714	4 (2)	4 ^R^	32 ^R^	128 ^R^	4 (2)	CMY-146	361
	DC11105	4 (2)	128 ^R^	32 ^R^	≥256 ^R^	4 (2)	NDM	405
	DC11403	4 (2)	16 ^R^	16 ^R^	16 ^R^	4 (2)	NDM-5	774
	DC11552	8 (4.1)	16 ^R^	16 ^R^	32 ^R^	16 (8.2)	KPC-2	15316
	DC11722	4 (2)	16 ^R^	16 ^R^	128 ^R^	8 (4.1)	CTX-M-199	167
	DC11723	8 (4.1)	64 ^R^	16 ^R^	128 ^R^	8 (4.1)	EC-15	167
	DC13314	4 (2)	128 ^R^	32 ^R^	256 ^R^	4 (2)	CMY-42	167
	DC14183	8 (4.1)	64 ^R^	16 ^R^	128 ^R^	4 (2)	CTX-M-15	405
	ATCC25922	4 (2)	0.01 ^S^	0.06 ^S^	0.08 ^S^	4 (2)	/	/
*K. pneumoniae*	FK4603	16 (8.2)	256 ^R^	64 ^R^	256 ^R^	16 (8.2)	/	ST11
	FK6299	16 (8.2)	≥256 ^R^	128 ^R^	≥256 ^R^	16 (8.2)	/	ST11
	FK6506	16 (8.2)	256 ^R^	128 ^R^	≥256 ^R^	16 (8.2)	/	ST11
	FK6625	16 (8.2)	256 ^R^	64 ^R^	≥256 ^R^	16 (8.2)	/	ST11
	FK6719	16 (8.2)	≥256 ^R^	128 ^R^	≥256 ^R^	16 (8.2)	KPC-2	ST11
	FK6741	16 (8.2)	256 ^R^	128 ^R^	≥256 ^R^	16 (8.2)	KPC-2	ST11
	FK6748	16 (8.2)	256 ^R^	128 ^R^	≥256 ^R^	16 (8.2)	KPC-2	ST11
	FK6818	8 (4.1)	256 ^R^	128 ^R^	≥256 ^R^	16 (8.2)	KPC-2	ST11
	FK6835	16 (8.2)	256 ^R^	64 ^R^	≥256 ^R^	16 (8.2)	KPC-2	ST11
	FK6845	16 (8.2)	256 ^R^	64 ^R^	≥256 ^R^	16 (8.2)	KPC-2	ST11
	FK6847	8 (4.1)	≥256 ^R^	128 ^R^	≥256 ^R^	16 (8.2)	KPC-2	ST11
	FK7153	8 (4.1)	≥256 ^R^	128 ^R^	≥256 ^R^	16 (8.2)	KPC-2	ST11
	FK7156	16 (8.2)	256 ^R^	64 ^R^	≥256 ^R^	16 (8.2)	KPC-2	ST11
	FK7787	16 (8.2)	256 ^R^	128 ^R^	≥256 ^R^	16 (8.2)	KPC-2	ST11
	FK7788	16 (8.2)	256 ^R^	64 ^R^	≥256 ^R^	16 (8.2)	KPC-2	ST11
	FK8078	8 (4.1)	256 ^R^	128 ^R^	≥256 ^R^	16 (8.2)	KPC-2	ST11
	FK8132	16 (8.2)	≥256 ^R^	128 ^R^	≥256 ^R^	16 (8.2)	KPC-2	ST11

^*a*^ MEM, meropenem; IPM, imipenem; ETP, ertapenem; S, susceptible; R, resistant.

^*b*^ Breakpoints (S–R): MEM (1–4 µg/mL), IPM (1–4 µg/mL), ETP (0.5–2 µg/mL).

^*c*^ The corresponding µM for µg/mL: MEM (4 µg/mL = 10.4 µM, 8 µg/mL = 20.8 µM, 16 µg/mL = 41.7 µM, etc); IPM (4 µg/mL = 13.3 µM, 8 µg/mL = 26.7 µM, 16 µg/mL = 53.4 µM, etc); ETP (4 µg/mL = 7.7 µM, 8 µg/mL = 15.4 µM, 16 µg/mL = 30.8 µM, etc).

**Fig 1:**
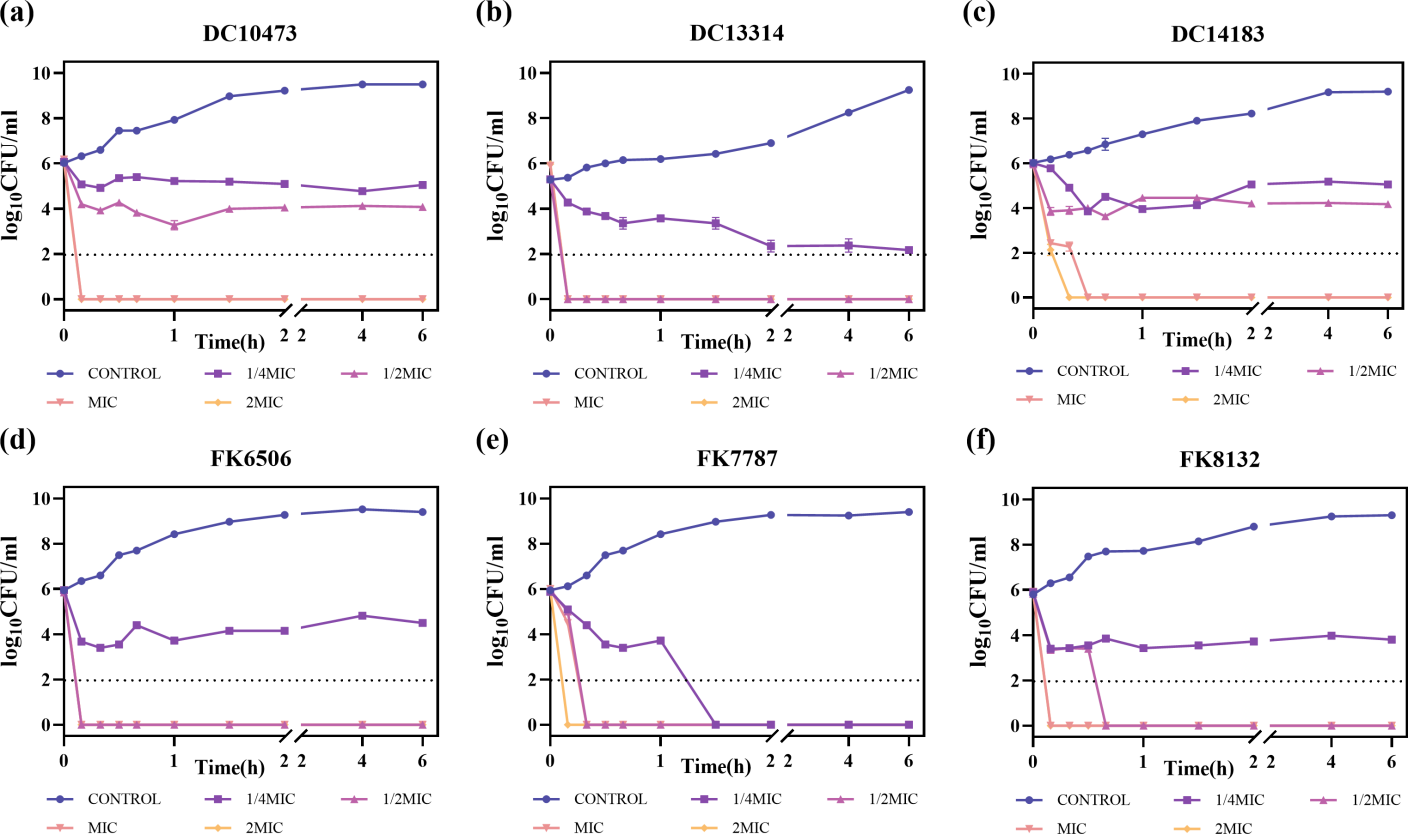
Time-killing curves of antimicrobial peptide Hs02 against carbapenem-resistant *K. pneumoniae* and *E. coli*. (**a–c**) Carbapenem-resistant *E. coli*, (**d–f**) carbapenem-resistant *K. pneumoniae*. Experiments were repeated three times.

The time-dependent killing assay of three strains of CRKP and CREC revealed that Hs02 can rapidly kill bacteria within 10–30 min at the MIC concentration. The MIC value of Hs02 against CRKP was relatively higher compared to CREC (Fig. 1a through f). However, at doses of 1/4 MIC and 1/2 MIC, Hs02 showed more potent bactericidal activity and a longer duration of inhibition against CRKP compared to CREC. In both MIC and 2 MIC conditions, Hs02 demonstrated significant bactericidal efficacy within a 6 h period.

### Hs02 inhibited and disrupted CRKP and CREC biofilms *in vitro*

**Fig 2:**
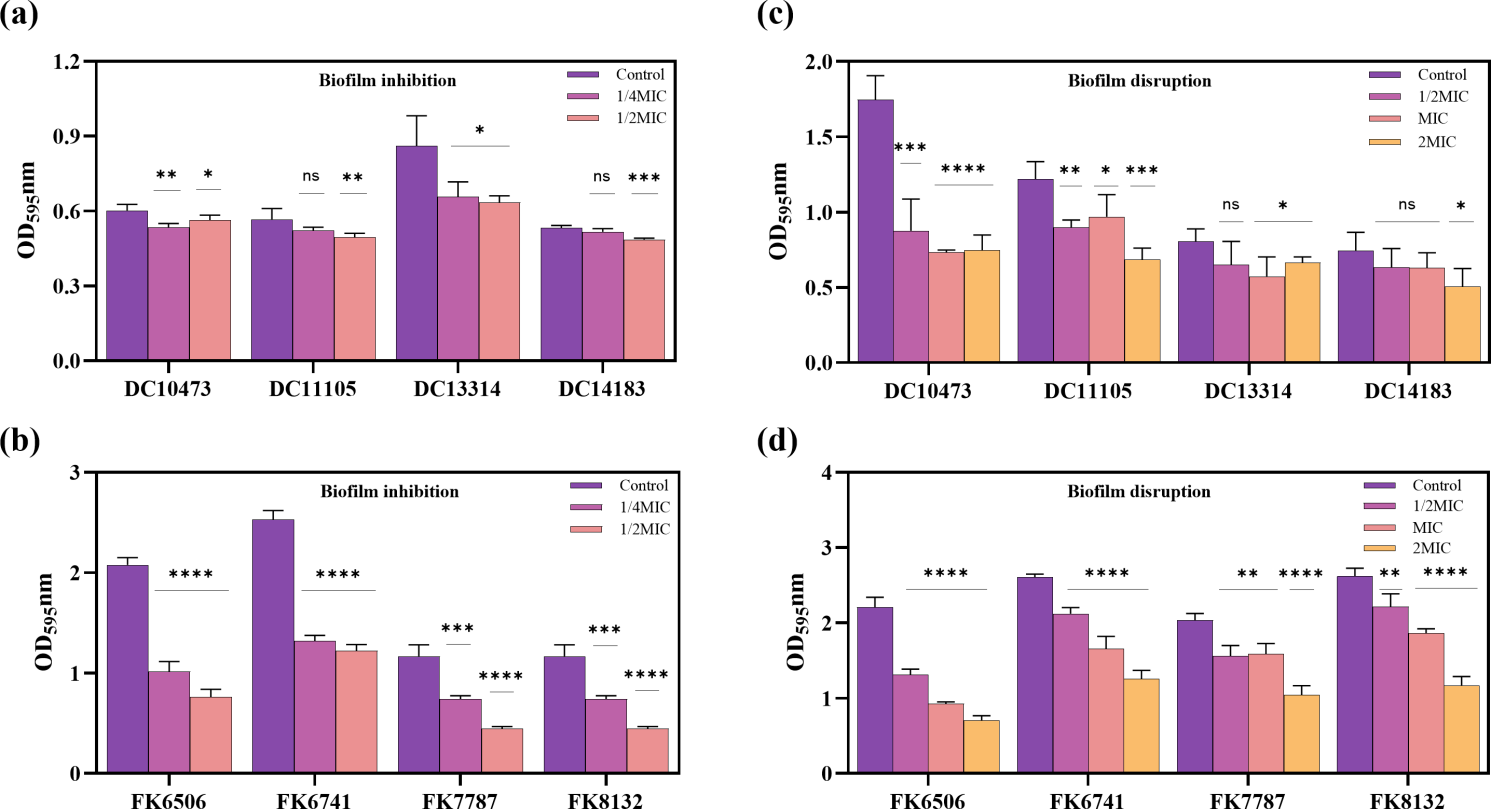
Anti-biofilm effect of Hs02. (**a, c**) Biofilm formation inhibition assays and biofilm disruption assays were performed on four *E. coli* strains. (**b, d**) Biofilm formation inhibition assays and biofilm disruption assays were performed on four *K. pneumoniae* strains. Experiments were performed with four biological replicates, and all data are presented as mean ± SD.

The ability of Hs02 to act on CRKP and CREC biofilms was investigated by crystal violet staining. Four strains each of CRKP and CREC were selected as experimental strains. In the biofilm inhibition and disruption assays, the incubation duration for *E. coli* was extended to 48 h due to its comparatively lower biofilm-forming capacity compared to *K. pneumoniae*. As depicted in Fig. 2a and b, biofilm production was decreased in a dose-dependent manner for all eight strains at subinhibitory concentrations. As illustrated in Fig. 2c and d, the same strains were utilized in the established biofilm disruption assay. At concentrations of 1/2 MIC, MIC, and 2 MIC, Hs02 effectively disrupted the established biofilms when compared to the control. These results suggest that Hs02 demonstrates anti-biofilm activity against CRKP and CREC.

### LPS is a target for the antimicrobial activity of Hs02

Predictive analysis suggested that Hs02, a cationic antimicrobial peptide, may show antimicrobial mechanisms similar to those of other cationic antimicrobial peptides. A common mechanism involves membrane disruption, where the positively charged peptide interacts with the negatively charged extracellular polysaccharides and LPS of the bacterial outer membrane (OM). After binding to the bacterial OM, the peptide’s hydrophobic properties facilitate its insertion into the bacterial cell membrane, leading to disruption of membrane permeability and integrity (24).

**Fig 3:**
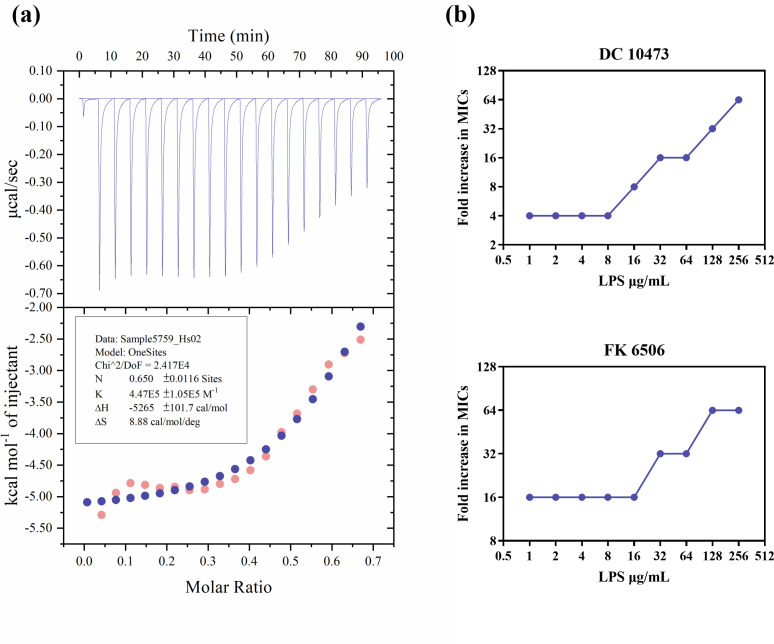
(**a**) The experimental data of LPS titration by Hs02 and the calorimetric titration curve for Hs02 binding to LPS, with the blue curve being the fitted curve of the pink curve. (**b**) Fold change in MIC values of DC10473 and FK6506 after the addition of exogenous LPS. Experiments were repeated three times.

To validate this hypothesis, isothermal titration calorimetry (ITC) was performed to analyze the thermodynamic parameters and binding affinity of Hs02 with LPS. As shown in Fig. 3a, the ITC results indicated an exothermic interaction. The interaction enthalpy (∆H) and entropy (∆S) were −5,265 ± 101.7 cal/mol and 8.88 cal/mol/deg, respectively, demonstrating a strong interaction and high binding affinity between Hs02 and LPS. To further confirm the binding ability of Hs02 with LPS, competition experiments were performed using varying concentrations of exogenous LPS. The MIC values of the two bacterial strains increased in a concentration-dependent manner with the addition of exogenous LPS, indicating that exogenous LPS competed with Hs02, thereby reducing the number of interactions between Hs02 and the bacterial cell membrane (Fig. 3b).

### Hs02 disrupted membrane integrity and induced reactive oxygen species production

**Fig 4:**
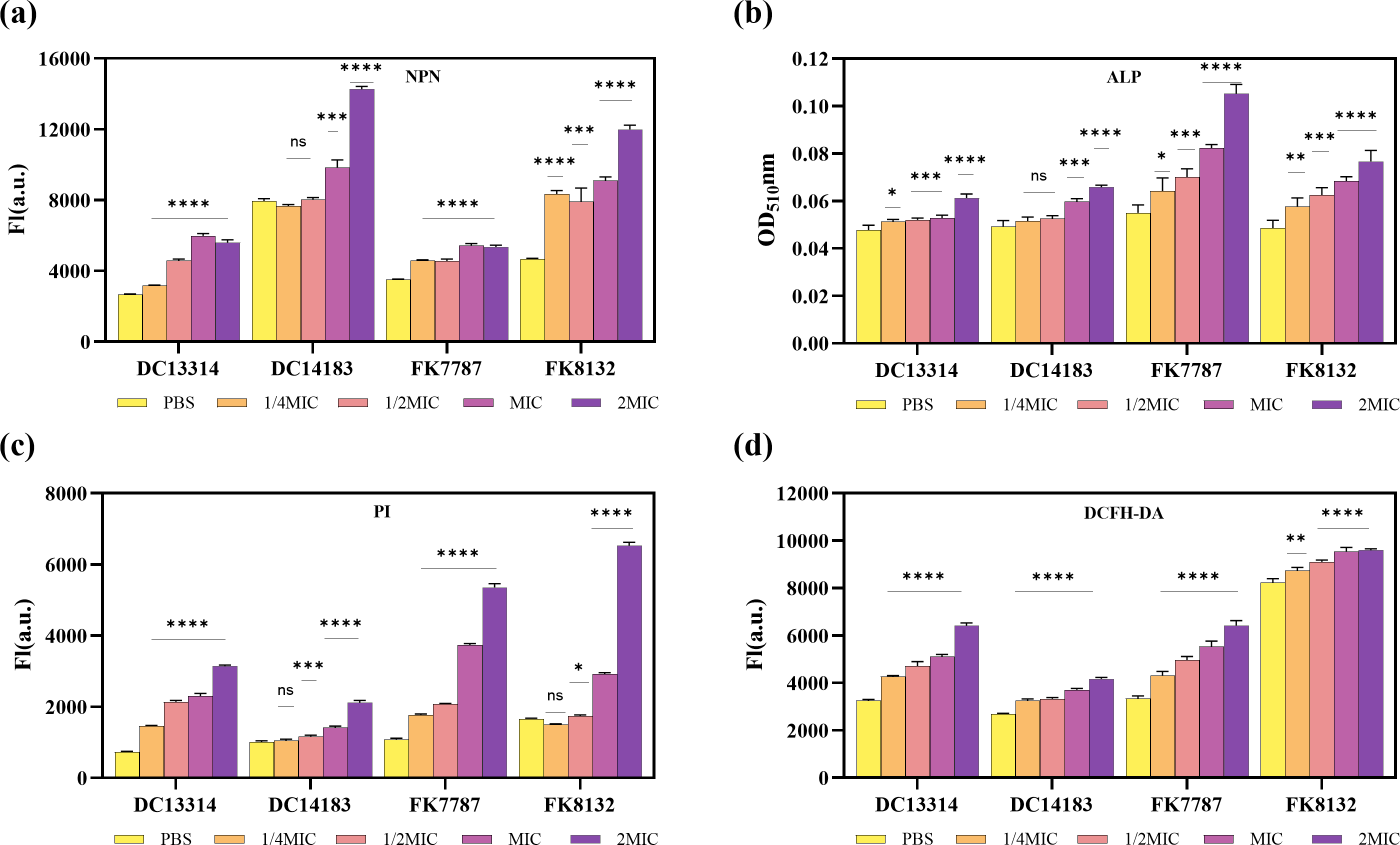
Antimicrobial mechanisms of Hs02. (**a**) OM perturbation was measured using the NPN assay. (**b**) OM permeability is determined by released periplasmic ALP. (**c**) IM permeability is determined by propidium iodide (PI) dye. (**d**) Quantification of reactive oxygen species (ROS) in different concentration treatments. Experiments were performed with four biological replicates, and all data are presented as mean ± SD.

Several tests were employed to examine the possible antibacterial mechanism of the antimicrobial peptide Hs02. The antibacterial mechanism experiment employed a set of consistent bacterial strains (FK8132, FK7787, DC13314, DC14183) to perform research. An initial study was focused on examining changes in membrane permeability. To evaluate the effect of Hs02 on the OM, a hydrophobic fluorescent dye (NPN [N-phenylnaphthalen-1-amine]) was used at different experimental doses. Small molecules of NPN are unable to traverse the lipid bilayers of the bacterial cell membrane when an intact OM is present. When OM is disrupted, NPN binds to OM and fluorescence is enhanced. As shown in Fig. 4a, Hs02 significantly increased OM permeability in a dose-dependent manner compared to the control group. The extent of Hs02-induced damage to the bacterial OM was further assessed using an alkaline phosphatase (ALP) leakage assay. The ALP, macromolecular proteins located in the periplasm of bacterial cells, leak out only when the OM is severely compromised. As depicted in Fig. 4b, a significant difference in ALP release was observed between the experimental and control groups, with the release being dose-dependent.

To assess the possible influence of Hs02 on changes in the permeability of the inner membrane (IM) of bacterial cells, the cells were exposed to propidium iodide dye (PI). The capacity of PI to penetrate an undamaged cell membrane is restricted since it can solely access the cytoplasm if the membrane is disrupted. After entering, PI attaches to DNA and subsequently produces fluorescence. The fluorescence intensity emitted by PI increased in proportion to the amount of Hs02, demonstrating that Hs02 has the potential to cause an increase in IM permeability (Fig. 4c).

Further investigation was performed to determine whether Hs02 could cause bacterial damage through mechanisms other than disrupting cell membrane integrity. The 2',7'-dichlorodihydrofluorescein diacetate (DCFH-DA) fluorescent probe, which is quenched in the extracellular environment, can be hydrolyzed by intracellular esterase, leading to the formation of DCFH. DCFH is then oxidized by reactive oxygen species (ROS) to produce the green fluorescent molecule DCF (25). The treatment group displayed a direct correlation between increasing drug concentration and fluorescence intensity, unlike the control group. This result confirms that Hs02 can also damage bacteria through the accumulation of intracellular ROS (Fig. 4d).

### Hs02 demonstrated non-toxicity at the experimental concentrations

**Fig 5:**
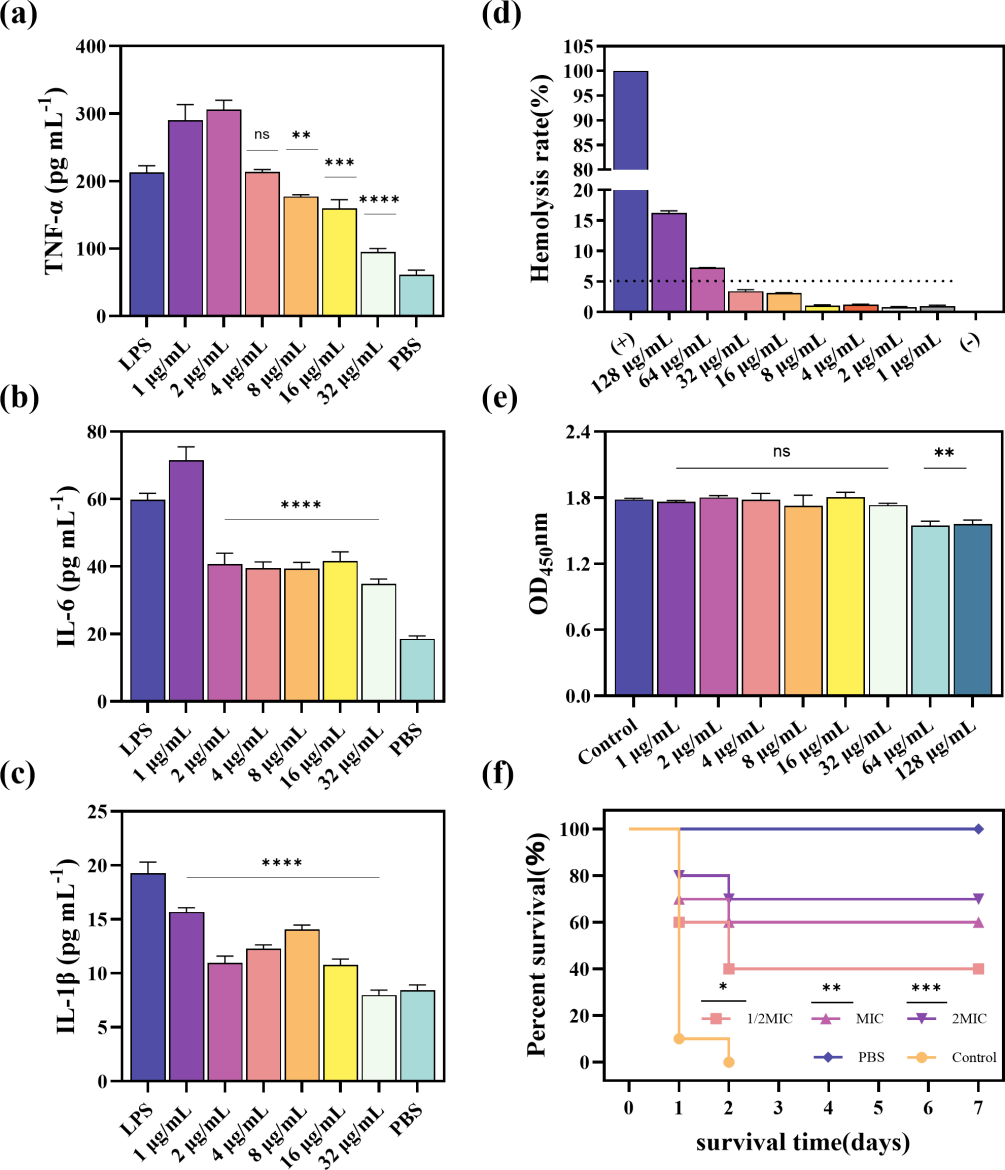
(**a–c**) Quantification of inflammatory cytokines TNF-α, IL-6, and IL-1β by enzyme-linked immunosorbent assay (ELISA). (**d**) Hemolytic activity of Hs02 at varying concentrations (1–128 μg/mL). Hemolysis rates of 5% or higher were considered positive. (**e**) Cytotoxic effects of different concentrations of Hs02 on RAW 264.7 mouse macrophages. Phosphate-buffered saline (PBS) was used as a control group. Each experiment was performed in triplicate, and all data are presented as mean ± SD. (**f**) The survival rate of *Galleria mellonella* larvae after Hs02 treatment. *E. coli* (DC14183) was used as the experimental strain. The significance analysis is between the treatment group and the control group. Experiments were repeated three times.

The hemolytic activity and cytotoxicity of antimicrobial peptides pose major challenges to the therapeutic use of these peptides. The hemolytic activity was evaluated using healthy mouse red blood cells, whereas the cytotoxicity was assessed using murine RAW264.7 cells. The antimicrobial peptide Hs02 showed a hemolytic rate of less than 5% on the red blood cells of healthy mice at a concentration of 32 µg/mL. Additionally, no significant difference in cytotoxicity was observed for 32 µg/mL Hs02 on murine RAW264.7 cells compared to the control group (*P* > 0.05). Based on the results of the erythrocyte hemolysis and cytotoxicity tests, all concentrations used in this study were within acceptable limits (Fig. 5d and e).

### Hs02 inhibited pro-inflammatory cytokine and improved survival in infected *Galleria mellonella* larvae

Bacterial infection induces endotoxin production, which stimulates macrophages to produce high levels of pro-inflammatory cytokines, including TNF-α, IL-6, and IL-1β. LPS, the main component of endotoxin, was used to stimulate RAW 264.7 cells at a concentration of 1 µg/mL, followed by treatment with various concentrations of Hs02. As shown in Fig. 5a through c, the results indicated that Hs02 significantly reduced TNF-α levels at concentrations between 8 and 32 µg/mL, while it lowered IL-6 and IL-1β levels at concentrations ranging from 2 to 32 µg/mL, compared to the control (LPS only). At a concentration of 32 µg/mL, the levels of TNF-α and IL-1β were reduced to those comparable to the PBS negative control.

To initially investigate the *in vivo* antimicrobial effectiveness of Hs02, the survival rates of *Galleria mellonella* larvae increased by 40%, 60%, and 70% when administered therapeutic doses equivalent to 1/2 MIC, MIC, and 2 MIC, respectively, compared to the control group (Fig. 5f). These findings suggest that Hs02 exhibits significant antibacterial activity in *Galleria mellonella* larvae.

## DISCUSSION

Currently, carbapenems are the most effective treatment for infections caused by specific ESKAPE bacteria. However, the increasing use of carbapenem in clinical settings has led to selection pressure, resulting in the emergence of CRKP and CREC strains (26).

*K. pneumoniae* and *E. coli* can form biofilms, where bacteria within these biofilms are often exposed to subinhibitory concentrations of antibiotics, increasing the risk of developing drug resistance. In addition to reducing antibiotic efficacy, biofilm formation can enhance quorum sensing between bacteria and impede phagocytosis by immune cells (27). The ability to rapidly kill bacteria, inhibit biofilm formation, and eliminate established biofilms is challenging (28).

In this study, Hs02 quickly eradicated *K. pneumoniae* and *E. coli* within 30 min, inhibited biofilm formation, and disrupted existing biofilms at subinhibitory concentrations of 1/4 MIC and 1/2 MIC. This rapid action was proposed to minimize the time available for bacterial adaptation to irreversible factors, thereby reducing the risk of developing bacterial resistance.

AMPs can regulate inflammatory cells and inhibit the release of pro-inflammatory cytokines, including TNF-α, IL-6, and IL-1β (29). However, their anti-inflammatory effects are not achieved through a single mechanism but involve multiple pathways, such as neutralizing LPS, competitively binding to LPS inhibiting its transport, and preventing LPS from binding to LPS binding protein. In this study, Hs02’s ability to neutralize LPS may be a crucial factor in its effectiveness at inhibiting the production of TNF-α, IL-6, and IL-1β by macrophages. During LPS-induced inflammatory responses, the activation of the NF-κB signaling pathway is pivotal. LPS triggers the MyD88-dependent cascade through TLR4, which activates IκB kinase. This kinase then phosphorylates and degrades the IκB protein, freeing NF-κB to translocate into the nucleus, where it promotes the expression of inflammatory cytokines like TNF-α and IL-1β (30, 31).

There are two primary models for understanding the antibacterial mechanism of AMPs: membrane targeting and targeting molecules in the envelope. Among these models, membrane destruction is the more prominent approach (32). Hs02, as a cationic molecule, carries a positive charge that allows it to interact with negatively charged LPS. This interaction is the first step in Hs02’s engagement with the bacterial cell membrane. Cationic AMPs like Hs02 are strongly attracted to the polar LPS layer through electrostatic interactions, resulting in the formation of multiple defects in the LPS layer. Simultaneously, upon binding to LPS, the hydrophilic groups of Hs02 may interact with the hydrophilic ends of phospholipids in the bacterial OM, facilitating its insertion into the phospholipid bilayer. Due to its amphipathic properties, the inserted hydrophobic groups repel the hydrophobic tails of the phospholipids, leading to membrane disruption, loss of membrane integrity, and increased intracellular ROS accumulation. Compared to antimicrobial agents that target metabolic activity, membrane-disrupting antimicrobials generally have a shorter bactericidal time, which may account for Hs02’s ability to rapidly kill bacteria within 30 min (33–35). The present study demonstrates that Hs02 has a dual capability: it effectively kills bacteria and neutralizes LPS, thereby inhibiting the expression of inflammatory cytokines.

The cytotoxicity and hemolytic activity of AMPs are critical factors for their *in vivo* application. Given the amphipathic properties of Hs02, there is a potential risk of membrane disruption to mammalian cell membranes (phospholipid bilayers). However, present *in vitro* hemolysis and cytotoxicity assay findings show that Hs02 demonstrates a certain degree of safety at experimental concentrations.

There are some limitations of the study. Firstly, the conclusions are based on *in vitro* experiments, with cytotoxicity and hemolysis results derived from RAW264.7 cells and mouse erythrocytes. Additionally, the use of a single mouse macrophage cell line (RAW264.7) to investigate the anti-inflammatory activity of Hs02 does not fully reflect the *in vivo* multi-cellular and multi-pathway anti-inflammatory mechanisms. While Hs02’s neutralization of LPS contributes to its anti-inflammatory activity, other potential immunomodulatory mechanisms cannot be ruled out. These limitations highlight the need for further *in vivo* studies to validate the systemic safety and anti-inflammatory efficacy of Hs02. Secondly, since antimicrobial peptides consist of multiple amino acid residues, optimizing these residues through computer prediction can help achieve an optimal balance between antimicrobial activity and safety (36–38).

### Conclusions

In conclusion, the antimicrobial peptide Hs02 rapidly kills CRKP and CREC, inhibits and disrupts their biofilms, and primarily targets and binds to LPS on the bacterial cell membrane. Hs02 also reduces the production of pro-inflammatory cytokines in mouse macrophages following LPS neutralization. These findings suggest that Hs02 has potential as a therapeutic candidate and a model for designing antimicrobial peptides against CRKP and CREC infections.

## MATERIALS AND METHODS

### Antimicrobial peptides and antimicrobial drugs

Hs02 was synthesized by Nanjing Yuanpeptide Biotechnology Co., Ltd. in Nanjing, China. The amino acid sequence of Hs02 was KWAVRIIRKFIKGFIS-NH_2_ (molecular weight: 1,961.44, https://aps.unmc.edu/AP/ APD ID: AP03104, PDB: 6MBM). The antibiotics (imipenem, meropenem, and ertapenem) used in this study were acquired from Wenzhou Kangtai Biotechnology Co., Ltd. (Zhejiang, China). Antimicrobial susceptibility testing was performed using Mueller-Hinton broth (MHB) from Thermo Fisher Scientific, USA.

### Bacterial isolates and growth conditions

The strain *E. coli* ATCC 25922 was purchased from the National Center for Clinical Laboratory for quality control purposes. A total of 16 CREC and 17 CRKP strains were obtained from the First Affiliated Hospital of Wenzhou Medical University, with duplicate strains from the same site in the same patient removed. CRKP and CREC are defined as the resistance of *Klebsiella pneumoniae* and *Escherichia coli* to at least one or more carbapenems. Species identification was performed using the VITEK MS IVD V3.2 (BioMérieux, France) identification system according to the manufacturer’s instructions. Strains were identified as “DCXXX” for *E. coli* and “FKXXX” for *K. pneumoniae*. MHB was used as the culture medium.

### Determination of MICs and MBCs

The antibacterial efficacy of Hs02, imipenem, meropenem, and ertapenem was evaluated against 33 clinical isolates of carbapenem-resistant strains using the broth microdilution method. The control strain used was *E. coli* ATCC25922 (39). Hs02 and antibiotics were diluted to concentrations ranging from 0.01 μg/mL to 256 μg/mL using serial dilutions in 96-well plates. Overnight-cultivated bacteria were standardized to a 0.5 McFarland standard and then diluted 1:100 in MHB. An aliquot of 100 µL of the diluted bacterial culture was combined with 100 µL of the diluted drugs solution, and the mixture was then incubated at 37°C for 15 to 20 h. The results of the MIC were interpreted following the criteria provided by the CLSI (M100, 34th). These guidelines define the MIC as the lowest concentration of an antibiotic that blocks the growth of bacteria, eliminating any small haze or turbidity. Each experiment was carried out separately and three times to ensure accuracy.

MBCs were determined by taking 100 µL of culture solution from the wells corresponding to the MIC concentration and the two adjacent higher concentrations. This solution was evenly spread on LB agar plates and incubated at 37°C for 16–20 h. The MBC was defined as the lowest concentration where no bacterial growth was observed on the LB agar plates.

### Time-dependent killing assays

A time-dependent killing assay was performed using a previously reported protocol with some modifications (40, 41). Various concentrations of Hs02 (1/4 MIC, 1/2 MIC, MIC, and 2 MIC) were introduced into MHB broth with 1 × 10^6^ CFU/mL of the test strain. The MIC values for the test strains selected in this study correspond to Table 1. The mixture was then incubated at 37°C, 200 rpm for 6 h. PBS served as the control. At specified time intervals (0, 10, 20, 30, 40, 60, 90, 120, 240, and 360 min), 20 µL of the bacterial suspensions were serially diluted 10-fold in saline and then plated onto LB agar plates. Four replicates were performed for each concentration. The plates were kept stationary at 37°C for 16–18. Colony counting was performed at concentrations where individual colonies were uniformly dispersed. The detection limit was set at two log10 CFU/mL.

### Biofilm inhibition and disruption assays

Biofilm inhibition experiments were performed as reported previously (42). Four strains of CRKP and CREC were chosen as the test strains. The experimental groups were divided into concentrations of 1/4 MIC and 1/2 MIC. A mixture of 100 µL of Hs02 solution and 100 µL of bacterial suspension was prepared in a 96-well microplate. The control received 200 µL of bacterial solution. *K. pneumoniae* cultures were incubated for 24 h and *E. coli* cultures for 48 h at 37°C. The excess medium was then removed, and the cultures were washed twice with PBS to remove planktonic bacteria, followed by drying. Each well was treated with 180 µL of 1% crystal violet (CV) dye (Solarbio Biotechnology LTD). The plates were incubated at a temperature of 37°C for 15 min, followed by two washes using PBS. Next, 150 µL of anhydrous ethanol was used to dissolve the bound CV attached. The measurement of absorbance was performed at a wavelength of 595 nm using a microplate reader (Multiskan FC; Thermofisher Scientific, USA). The experiment was repeated thrice.

The same strains were employed in both the biofilm disruption and biofilm formation inhibition assays. A 200 µL aliquot of bacterial suspension was inoculated into 96-well plates and incubated at 37°C for 24 h for *K. pneumoniae* and 48 h for *E. coli*. Following incubation, the wells were rinsed with PBS to remove planktonic bacteria. Following this, 200 µL of drug solutions at various concentrations (1/2 MIC, MIC, 2 MIC) were added, with 200 µL of drug-free broth serving as a control. The procedures for crystal violet staining, elution, and absorbance measurement in the biofilm disruption assay were consistent with those used in the biofilm inhibition experiment.

### ITC

The experiment was performed as described previously with slight modifications (43). Briefly, LPS *E. coli* O55:B5 was vortexed in PBS for 5 min before use. The experimental instrument used was the Malvern MicroCal VP-ITC from the UK. A 500 µM LPS solution was used as the titrant, and 100 µM Hs02 was used as the titrate. The titration parameters are listed in Fig. S1. The ITC data were analyzed using the one-site binding model in the MicroCal Origin software package to determine the equilibrium association constant (Ka), binding stoichiometry (n), and enthalpy change (∆H). The entropy change (∆S) and Gibbs free energy (∆G) were calculated using the equations ∆G = −RTlnKa and ∆S = (∆H − ∆G)/T, respectively.

### Effect of exogenous LPS on the activity of Hs02

The effect of adding exogenous LPS on the antibacterial activity of Hs02 was assessed following previous studies (44). Briefly, bacterial suspensions at 1.0 × 10^6^ CFU/mL (DC10473, FK6506) were co-cultured with LPS (0 to 256 µg/mL) and Hs02 (0 to 64 µg/mL) in sterile 96-well plates for 18–20 h at 37°C. The MIC values of Hs02 in the presence of exogenous LPS were then determined using the checkerboard dilution method.

### Membrane permeability assays and ROS detection

Four specific strains (DC13314, DC14183, FK7787, FK8132) were used in membrane permeability tests and (ROS) detection. The strains were incubated at 37°C with shaking for 6 h, then washed three times with PBS, and the absorbance was measured at OD_600_ (0.3–0.5). The OM and IM permeability was evaluated using the hydrophobic fluorescent probe 1-N-phenyl naphthylamine (NPN, Aladdin, Shanghai, China) and propidium iodide (PI, Yuanye, Shanghai, China), following a previously reported protocol with some modifications (45).

The ALP assay was performed using a previously established protocol with some modifications (46). A 1 mL of bacterial suspension was aliquoted into separate Eppendorf tubes. Each tube received different concentrations of Hs02 (1/4 MIC, 1/2 MIC, MIC, 2 MIC), with a control group treated with PBS. The tubes were incubated at 37°C for 4–6 h. Following incubation, the ALP detection reagent (Solarbio, Beijing, China) was added, and the fluorescence was measured at 510 nm to quantify ALP activity.

Intracellular levels of ROS were measured using the Reactive Oxygen Species Assay Kit from Beyotime Biotechnology (Solarbio, China) following the provided instructions (47). Briefly, 1 µL of DCFH-DA (10 µM) was added to 1 mL of bacterial culture and incubated at 37°C for 20–30 min. The culture was then rinsed three times with PBS, suspended in PBS to completely remove the fluorescent probe, and treated with Hs02 at concentrations of 1/4 MIC, 1/2 MIC, MIC, and 2 MIC, with PBS serving as the control. The culture was incubated at 37°C for 30 min. Fluorescence intensity was measured using a multifunctional microplate reader with excitation and emission wavelengths of 488/525 nm.

### *In vivo* infection model of *Galleria mellonella* larvae

The *in vivo* effectiveness of Hs02 was evaluated using a *Galleria mellonella* larvae infection model (48). DC14183 was selected as the experimental strain, and *Galleria mellonella* larvae weighing 200 to 300 mg, displaying a milky white color, and vigorous movement were used. This suspension was diluted to a concentration of 1.5 × 10^7^ CFU/mL. A volume of 10 µL of the bacterial suspension was injected into the untreated group, with PBS serving as the negative control. One hour after bacterial injection, the treatment group received 10 µL of Hs02 at different doses (1/2 MIC×7, MIC×7, 2 MIC×7). The increased dose accounts for the dilution effect upon injection into the *Galleria mellonella* larvae. Observations continued for 7 days, with the survival rate recorded every 24 h. *Galleria mellonella* larvae were considered deceased if they turned black and were unresponsive to stimuli.

### Hemolytic activity and cytotoxicity assays

In this experiment, 5 to 6-week-old female BALB/c mice from Charles River, Hangzhou, China, were used. To obtain plasma, 3 mL of fresh whole mouse blood was collected and centrifuged at 1,000 × *g* for 10 min at 4°C (49). Animal Welfare and Ethics criteria and was approved by the Zhejiang Association for Science and Technology (SYXK [ID: SYXK (Zhejiang) 2018-0017]). Following three PBS washes, the RBC suspension was diluted with regular saline to provide a 10% RBC suspension. To get a final concentration of Hs02 (1–128 μg/mL) and 5% RBC suspension, 300 µL of 10% RBC suspension was mixed with different concentrations of Hs02 (2–256 μg/mL). Following a 1-h incubation at 37°C and 5 min centrifugation at 1,000 × *g*, the supernatant was transferred to 96-well plates. Hemolysis was assessed by measuring absorbance at 540 nm. PBS was used for the negative control group, while 0.1% Triton X-100 served as the positive control. The experiment was performed in triplicate. Hemolysis rate (%) = (ODtest – ODnegative)/(ODpositive – ODnegative).

The safety of Hs02 was evaluated using RAW 264.7 cells according to a previously established method (50). Mouse mononuclear macrophage RAW 264.7 cells (1–2 × 10^5^ cells/well) were seeded in 96-well plates and incubated at 37°C for 10 h. Next, 10 µL of Hs02 was added to achieve final concentrations ranging from 1 to 128 µg/mL, and the cultures were incubated for an additional 24 h at 37°C with 5% CO_2_. After a 2 h incubation with 10 µL of Cell Counting Kit-8 (CCK-8) solution (NCM, Suzhou, China) at 37°C, absorbance was measured at 450 nm to assess cytotoxicity. The experiment was performed in triplicate.

### Measurement of cytokine concentrations by ELISA

RAW 264.7 cells were stimulated by adding 1 µg/mL LPS, following the established protocol (51). With varying quantities of Hs02 applied and incubated for 4 h, the supernatant was collected. TNF-α, IL-6, and IL-1β levels were quantified using an enzyme-linked immunosorbent assay (ELISA) kit (J&L Biological), following the manufacturer’s instructions.

### Statistical analysis

Statistical analyses were performed using GraphPad Prism 10.2.0 software. The data were represented as mean ± SD. Each experiment was performed in triplicate. Statistical significance was assessed using either a two-sample *t*-test or one-way analysis of variance followed by Tukey’s multiple-comparison test. The survival curves were analyzed using the Log-rank (Mantel-Cox) test. Results were considered statistically significant at *P* < 0.05 (*), *P* < 0.01 (**), *P* < 0.001 (***), and *P* < 0.0001 (****), while “ns” indicates not statistically significant.

## SUPPLEMENTAL MATERIAL

Figure S1 spectrum.01050-24-s0001.docx Instrument parameter settings for the isothermal titration test.

ASM does not own the copyrights to Supplemental Material that may be linked to, or accessed through, an article. The authors have granted ASM a non-exclusive, world-wide license to publish the Supplemental Material files. Please contact the corresponding author directly for reuse.
