# Supplementary material for: Antimicrobial peptide Hs02 with rapid bactericidal, anti-biofilm, and anti-inflammatory activity against carbapenem-resistant Klebsiella pneumoniae and Escherichia coli
Source: Microbiol Spectr. 2024 Dec 3;13(1):e01050-24. doi: 10.1128/spectrum.01050-24 (PMC11705930; doi:10.1128/spectrum.01050-24)
Supplement: Figure S1 — Instrument parameter settings for the isothermal titration test. [file spectrum.01050-24-s0001.docx]

**Supplemental figure
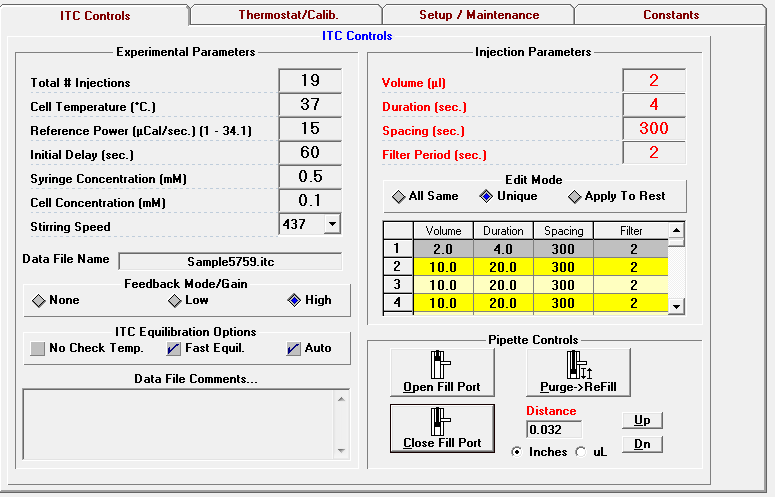
**

**Supplementary Figure 1.** The parameters set for the isothermal titration experiments in this study were conducted using the Malvern MicroCal VP-ITC from the UK.
